# Prolonged Non-metabolic Heart Rate Variability Reduction as a Physiological Marker of Psychological Stress in Daily Life

**DOI:** 10.1007/s12160-016-9795-7

**Published:** 2016-05-05

**Authors:** Bart Verkuil, Jos F. Brosschot, Marieke S. Tollenaar, Richard D. Lane, Julian F. Thayer

**Affiliations:** 1 Clinical Psychology and the Leiden Institute of Brain and Cognition, Leiden University, Wassenaarseweg 52, 2333 AK Leiden, The Netherlands; 2 The University of Arizona, Tucson, AZ USA; 3 The Ohio State University, Columbus, OH USA

**Keywords:** Heart rate variability, Perseverative cognition, Worry, Emotional awareness, Physical activity, Metabolic

## Abstract

**Background:**

Prolonged cardiac activity that exceeds metabolic needs can be detrimental for somatic health. Psychological stress could result in such “additional cardiac activity.”

**Purpose:**

In this study, we examined whether prolonged additional reductions in heart rate variability (AddHRVr) can be measured in daily life with an algorithm that filters out changes in HRV that are purely due to metabolic demand, as indexed by movement, using a brief calibration procedure. We tested whether these AddHRVr periods were related to worry, stress, and negative emotions.

**Methods:**

Movement and the root of the mean square of successive differences (RMSSD) in heart rate were measured during a calibration phase and the subsequent 24 h in 32 participants. Worry, stress, explicit and implicit emotions were assessed hourly using smartphones. The Levels of Emotional Awareness Scale and resting HRV were used to account for individual differences. During calibration, person-specific relations between movement and RMSSD were determined. The 24-h data were used to detect prolonged periods (i.e., 7.5 min) of AddHRVr.

**Results:**

AddHRVr periods were associated with worrying, with decreased explicit positive affect, and with increased tension, but not with the frequency of stressful events or implicit emotions. Only in people high in emotional awareness and high in resting HRV did changes in AddHRVr covary with changes in explicit emotions.

**Conclusions:**

The algorithm can be used to capture prolonged reductions in HRV that are not due to metabolic needs. This enables the real-time assessment of episodes of potentially detrimental cardiac activity and its psychological determinants in daily life.

## Introduction

Low heart rate variability (HRV) is a risk factor for a wide range of pathogenic states and for mortality [1]. For example, low HRV is predictive of insulin resistance [2] and inflammation [3], and has been found to predict the development of cardiovascular disease [4] and of renal dysfunction [5] in initially healthy people. Given the prognostic value of HRV, research into the antecedents, correlates, and consequences of low HRV levels is flourishing. Low HRV is especially believed to be detrimental for health when it is sustained for a prolonged period of time, for example, when resting levels are low. Low resting levels of HRV have been attributed to genetic factors [6] and to aging, as HRV gradually declines throughout the life span [7].

Two other major determinants of prolonged periods of low HRV—ones that we can potentially influence—are psychological stress and physical activity. With respect to stress, recurrent or prolonged periods of psychological stress are associated with prolonged decreases in HRV, and such prolonged states of low HRV might eventually contribute to lower resting levels of HRV, via a process called allostasis [8]. Physical activity is also known to temporarily reduce HRV levels, but it is generally believed that physical activity promotes somatic health and that aerobic exercise increases resting levels of HRV [9]. Taking physical activity into account when examining the detrimental impact of psychological variables on HRV is therefore critical. Yet, many studies that examine the association between HRV assessed in daily life (e.g., using 24-h Holter monitoring) and health outcomes usually do not measure physical activity, and thereby yield biased estimations of (A) the effects of psychological variables on HRV or (B) the impact of HRV on disease outcomes (see [10] for a similar argument). To illustrate that physical activity is largely ignored in ambulatory studies on HRV, we inspected the literature on ambulatory assessed HRV published in 2014 (keyword profile: (ambulatory OR 24-hour) and HRV) in Pubmed. Of the 23 full text papers that we inspected, in only 2 studies [11, 12] physical activity was measured using an accelerometer and physical activity was taken into account. In other ambulatory studies on stress and HRV, periods with high levels of physical activity were simply excluded [13–15], but this implies that stress and worries can only be measured when people are not moving, which limits the very advantage that ambulatory studies have on laboratory studies. In the present study, we examine a new approach to studying the link between psychological stress, physical activity, and HRV in daily life. In this approach, the variable of interest is not the HRV level per se, but those periods in daily life wherein HRV is persistently reduced and the reduction in HRV cannot be ascribed to physical activity.

The idea that physical activity should be taken into account when studying the link between stress and cardiac activity is not new. In the 1970s, researchers made clear that a distinction should be made between the effects of psychological stress and physical activity on heart rate (HR), and the changes in HR due to physical activity were considered adaptive, “context-appropriate,” or “metabolically warranted,” whereas those due to psychological stress were believed to be excessive and detrimental for health [16, 17]. Laboratory studies have indeed shown that during physically demanding tasks, cardiac activity is in line with the level predicted by oxygen consumption (an index of metabolic needs), but that psychological stress is associated with cardiac activity that exceeds the level predicted by oxygen consumption [17, 18].

Only a few attempts have been made to study this so-called additional heart rate activity or non-metabolic heart rate activity in daily life. In these ambulatory studies, additional HR was detected when HR increased by minimally 3 beats per minute compared to the previous 3 min, with no (or only a small) accompanying increase in physical activity [19]. Additional HR was found to be associated with emotional arousal [19–23].

Person-specific calibration methods have also been used, where the relation between metabolic need (oxygen consumption or movement) is related to cardiac activity during an initial calibration procedure. Data derived from this calibration are then entered into a person-specific regression model, which is used to determine when subsequent cardiac activity is additional to the activity predicted by this regression model. That is, cardiac activity is said to be “additional,” when it is above the level of what is being predicted by the regression model derived from a calibration phase. This procedure is used mostly in lab situations where oxygen consumption can be measured [18, 24, 25], or by using measures of respiration in daily life [26], but this requires invasive and expensive measurements that are not commonly available. Although using oxygen consumption and, to a lesser extent, respiration parameters could be considered the gold standard, metabolic needs can also be reliably estimated by movement [19]. A calibration approach based on movement data was described by Johnston [27]. He described a method to estimate additional HR in daily life on the basis of HR data and (EMG) movement data obtained from the thigh, during one calibration day. Data from this day were then used to build person-specific regression models, which were used on the subsequent day to detect periods of additional HR. However, one limitation of using a whole measurement day to derive regression models is that the regression model will be influenced by periods where stress-related additional HR is present (e.g., low movement, but unexpectedly high HR during calibration). Models that are influenced by these periods are prone to filter out those periods that we are interested in, and indeed Johnston found only a few periods of additional HR [27]. A short calibration period might therefore be preferred, which also can be integrated more easily into new study protocols compared to a whole additional calibration day.

In the present ambulatory study, we aimed to apply the idea of measuring non-metabolic activity to HRV and to detect much longer periods of additional reductions in HRV (AddHRVr), via a short calibration procedure. An important improvement to previous research is that we focus on testing a method to detect *prolonged* periods of additional cardiac activity. According to the prolonged activation hypothesis [28], HRV must be low for a sustained period of time in order to be detrimental for health. In contrast, sudden quick changes in HR(V)—as captured with the system developed by Myrtek and colleagues—could reflect adaptive emotional responding to changing environmental demands. In the present study, we tested whether periods of 7.5 min (15 epochs of 30 s) wherein HRV was continuously reduced below the levels that are metabolically needed were associated with psychological stress. Although other periods can be chosen, we here aimed to provide a proof-of-principle, that is, whether it is possible to capture non-metabolic reductions in HRV using the algorithm that we provided. Moreover, periods >5 min are long enough to represent mental states that are more than just short bursts of cognitive or emotional reactivity (e.g., sudden surprises, short irritations), which are unlikely to have health consequences. Another advantage to the method by Myrtek and colleagues is that we used person-specific algorithms to distinguish between metabolically warranted reductions in HRV and non-metabolic reductions in HRV, based on acceleration. For each participant, we obtained the relation between movement/acceleration and HRV during a calibration phase, and used this information to estimate whether prolonged non-metabolic reductions in HRV were present during the subsequent day.

In this study, we tested whether by using person-specific relations between movement and HRV, we were able to detect episodes of AddHRVr in daily life. We predicted that during hours wherein AddHRVr episodes were present, psychological stress would be enhanced, compared with hours without AddHRVr episodes. “Stress” was measured in several ways. First, it was measured as the occurrence of stressful events during the previous hour. Additionally, people are not continuously facing stressful situations in their daily lives, but they can recreate those situations in their minds, for example in anticipation of stressful events [29]. We therefore also assessed the occurrence of worry episodes, which are also associated with decreases in HRV. Furthermore, we examined whether self-reported emotions would be associated with the occurrence of AddHRVr episodes. More specifically, we expected that hours containing an AddHRVr episode would be associated with increased levels of negative affect and decreased levels of positive affect. Yet, we recently proposed that stress-related physiology might also be affected by mental processes of which we are not consciously aware [30–32]. We therefore also administered an ambulatory version of the Implicit Positive and Negative Affect Test [33].

Finally, we examined the extent to which the relation between our measures of stress and AddHRVr would be affected by individual differences in (A) levels of emotional awareness and (B) resting HRV. We did this because not every person is able to report on their own emotions and feelings of stress. According to the neurovisceral integration model [32, 34], both levels of emotional awareness and resting HRV levels express the extent to which people are able to recruit cortical areas in the brain, particularly in the medial prefrontal cortex, to describe their emotions. Low levels of emotional awareness, as well as low resting levels of HRV could predispose people to experience sustained periods of low HRV but also to not consciously report stress. We specifically predicted that, in people high in emotional awareness or high resting HRV, hours with AddHRVr episodes would be associated with increased explicit negative emotions and reduced positive emotions.

All in all, we here examined whether calculating AddHRVr in daily life would be feasible when using a short calibration procedure, and whether periods wherein AddHRVr episodes were present would be associated with psychological stress, especially in people who are able to reflect on their emotions as indexed by scores on the Levels of Emotional Awareness Scale and levels of resting HRV.

## Method

### Participants

Fifty-one students participated in a study on “emotions in daily life” [35] and were recruited at Leiden University. The sample consisted of healthy students that either were paid or received course credits for their participation. Ethical approval was obtained from the local ethical board.

### Procedure

After providing consent, participants were asked to report on cardiovascular and other medical problems. They also completed the Physical Activity Readiness Questionnaire [36] on which affirmative answers indicate a risk of medical problems that could occur during physical exercises. Participants subsequently engaged in a laboratory session during which emotional awareness was measured. Thereafter, they were instructed to put on the ECG and acceleration monitor, and completed a calibration session, consisting of the following phases: *rest* (sitting in front of a computer while watching a relaxing video, 3 min), *standing* (standing in front of the computer while conducting a simple counting task to prevent boredom/worrying, 3 min), *lying down* (on an examination table, 3 min), *cycling* (on an ergometer, in a fixed rhythm (75 bpm), 3 min), and *walking* up and down the stairs (168 steps in total). Data obtained during this calibration session were used afterwards to determine AddHRVr episodes. After the calibration, participants were instructed on how to use the smartphone and the ECG belt and a follow-up meeting was planned for 24 h later. In addition to the ECG assessment, participants also took saliva samples at five times during the day, to study changes in cortisol levels (data pertaining to the five cortisol assessments have been published elsewhere [35]). After the 24-h period, participants returned to the lab, were debriefed, and received course credits or a monetary reward for their participation.

### Instruments

#### *Heart Rate Variability and Acceleration*

The ecgMove sensor (Movisens, Gmhb, Karlsruhe, Germany) was used to record raw ECG as well as three-axis accelerometry signals. The EcgMove is attached to a chestbelt and worn at the base of the sternum and measures cardiovascular activity at 1024 Hz through two electrodes connected to the belt. After obtaining the ECG, this signal was further processed in Movisens Data-Analyzer software. The software used an automated algorithm to detect artifacts and processes the raw interbeat intervals into 30-s averages of the root of the mean square of successive differences (RMSSD) of the interbeat intervals. The ecgMove chestbelt also measures acceleration through a three-axial acceleration sensor with a sample rate of 64 Hz. The raw acceleration data (measured in g) is averaged over 30-s periods, from which mean acceleration per 30-s epoch was derived using the Movisens Data-Analyzer software. Resting levels of RMSSD were calculated as the mean RMSSD during the 3-min rest/sitting phase of the calibration.

#### *Sampling*

Motorola Razr smartphones were used for administering the psychological tests. MovisensXS software was used to program the smartphones.

#### *Stressful Events and Worry Episodes*

To capture stressors and worry episodes during the day, participants were asked to report these instances hourly on their smartphone between 10 AM and 10 PM. If they had experienced a stressor or a worry episode during the previous hour (“no” vs “yes”), participants are asked to estimate the timing of the event (<5 min ago, 5–15 min ago, 15–30 min ago, 30–45 min ago, 45–60 min ago, >60 min ago) as well as rate the duration of this event (<5, 5–15, 15–30, 30–45, 45–60, >60 min). Additionally, they are also asked to rate the intensity of the stressor on a five-point Likert scale ranging from “not at all” to “extremely.”

#### *Explicit Affect*

Participants were asked to rate the level of anger, happiness, sadness, and tenseness—as experienced since the last assessment—on a six-point Likert scale ranging from “not at all” to “extremely.” Participants’ scores on the anger, sadness, and tenseness subscales were averaged into one “negative affect” subscale. The happiness item served as the measure of “positive affect.” For the negative affect subscale, reliability was calculated following [37]. Between-person reliability for all three items, across all assessments, (RKF) was .97.

#### *Implicit Affect*

To measure implicit affect, the Implicit Positive and Negative Affect Test [33] was used. In the IPANAT, each hour a different nonsense word (e.g., “lawuk”) was presented to the participants and they were required to rate the extent to which this nonsense string expressed each of the following six emotions: happiness, being energetic, merriness, irritation, sadness, and tension. Participants are led to believe that the test is about finding onomatopoeias, and it is assumed that the ratings that they make actually express the amount of implicit affect. The assumption is that the participants respond in accordance with their current affective state, without being fully aware of the construct being measured. Participants provided their ratings on a scale from 1 (not at all) to 6 (very much). The IPANAT has high internal consistency (Cronbach’s *α* = .81) and test-retest reliability at 1 week ranged between .72 for positive affect and .76 for negative affect items [33]. These figures generally hold across different cultures [38]. In the current study, the average reliability for the positive affect scale (for all three items, across all assessments) was RKF = .96. For the negative affect scale, RKF was .94 [37].

Several studies have examined the criterion-related validity of the IPANAT [33, 35, 39]. Scores on the IPANAT subscales fluctuate in response to emotional pictures and recall of negative memories [33, 40]. Preliminary evidence for its convergent validity was demonstrated by the finding the positive and negative affect scores on the IPANAT were correlated with the amount of completed positive and negative fragments on an emotional word fragment completion task (*r*s = .20 and .22), another implicit test—which measures the amount of emotional information that is automatically (and thus implicitly) retrieved from memory. With respect to its predictive validity, in contrast to explicit affect ratings, the IPANAT subscales were related to cortisol levels [33, 35] and slow cardiovascular recovery after stress [39]. As these associations were largely independent of explicitly rated affect scores, this strongly suggests that the IPANAT measures affect beyond that which is consciously self-reported. In this line, scores on the IPANAT are only moderately associated with explicit ratings of affect (*r*s = .18–.33 [33]), thereby suggesting sufficient discriminant-related validity. Finally, most participants (0–.14 %) are not able to guess the real purpose of the IPANAT [33], adding to the idea that it is an indirect measure of affect that can explain variance in stress-related physiological activity beyond self-reports.

#### *Levels of Emotional Awareness*

The Levels of Emotional Awareness Scale developed by Lane et al. [41] was used to assess emotional awareness. The Dutch version was used [42]. Higher scores on the Levels of Emotional Awareness Scale are associated with greater emotion recognition ability [43, 44]. Scores on the Levels of Emotional Awareness Scale correlate positively with empathy ability [45], the perception of emotions in stories of the Multifactor Emotional Intelligence Scale [46] and the Range and Differentiation of Emotional Experience Scale [47]. Intratest homogeneity (*r* = .92; [43]), inter-rater reliability (Cronbach’s *α* = .88; [40]), and test-retest reliability at 4 weeks (*r* = .79; [48]) are high.

On the Levels of Emotional Awareness Scale, participants are presented with 10 scenarios and for each scenario they report how they would feel as the protagonist of each scene, and how the other person would feel. For example: “A loved one gives you a back rub after you return from a hard day’s work. How would you feel? How would your partner feel?”. For presenting the scenarios and collecting the answers, E-Prime was used. After each prompt, the participants were asked to type their answer into a provided text box. Each item received two scores (for self and other) between 0–5. The highest score of each item was added to generate the total score (max. 50). Two student raters scored the typed answers manually, and mainly used a word list with example words. In case a mentioned word could be assigned to more than one level (e.g., “ontspannen”—relaxed, level 1 or 3 were possible), the Levels of Emotional Awareness Scale manual [42] was consulted. If a word could not be found in the list, the raters used a synonym as instructed by the Levels of Emotional Awareness Scale manual. After both raters had scored all cases individually, they discussed their differences together with their supervisors (BV and MT) until they reached consensus to get a combined total score, which was used in the analysis. The intraclass correlation was .97.

#### *Additional HRV Detection Algorithm*

For each participant, an inverse regression curve was fitted on the data obtained during the calibration phase (Eq. 1). Graphical inspection of the data and parameters of explained variance (*R* ^2^) showed that inverse models provided a better fit for the data than linear models.

1 $$ \mathrm{Expected}\kern0.5em {\mathrm{RMSSD}}_{ij}=B{0}_i+\frac{B{1}_i}{{\mathrm{Acceleration}}_{ij}} $$

In doing so, we obtained *B*0_*i*_ (the intercept), which indicates the RMSSD value while no acceleration is present, and *B*1_*i*_ (slope), indicating the change in RMSSD due to acceleration. We also calculated the standard error of the mean of RMSSD during the calibration phase (SE).

Subsequently, the expected level of RMSSD was calculated for each individual (*i*), for each epoch (*j*), on the basis of the acceleration data obtained during the ambulatory phase of the study. Whenever the *actual* RMSSD level was two times the SE below the expected RMSSD level, this epoch was tagged as an “additional HRV decrease epoch.” (Thus, the threshold for detecting additional decreases in RMSSD was defined as follows: Threshold = Actual RMSSD − 2 × SE RMSSD_calibration_). When at least 15 of such epochs (i.e., 7.5 min) occurred subsequently and consecutively, the presence of an AddHRVr episode within that specific hour was detected. Finally, since stress measures were taken over whole hours, AddHRVr was also expressed in terms of hours. That is, hours with at least one episode of AddHRVr were coded as 1, hours without AddHRVr were coded as 0.

### Statistical Analysis

Given the hierarchical structure of the data, hourly assessments were nested within participants, and multilevel regression analyses were used (intraclass correlations for the affect measures were *r*s >.30, indicating clustering of the data within participants). Intercepts were allowed to vary between participants. Random slopes were omitted from the models as these led to convergence problems or did not improve model fit.

No additional autoregressive error covariance structures were modeled, as in most models no autocorrelation between the residuals was apparent. Only in the model predicting explicit negative affect, there was a small correlation between the errors (at lag 1; *r* = .13), but fitting an additional autoregressive error covariance structure did not reduce this.

AddHRVr was the independent variable (hours with AddHRVr episodes versus hours without AddHRVr episodes) and the ratings of explicit and implicit affect were the dependent variables (cf. [19]). We were particularly interested in the within-person association between AddHRVr and the dependent variables. We therefore created two variables, to be able to separate the within-persons association between AddHRVr and the dependent variables from the between-persons association between AddHRVr and the dependent variables [49]: for each participant, a stable (level 2) between-persons mean of the number of AddHRVr episodes was calculated (AddHRV-between), as well as the deviations from this mean, which represented the within-person change (AddHRV-within). If no within-person association between AddHRVr and a dependent variable was observed in the random intercept multilevel models, the between-person association was examined in the aggregated data using Pearson correlations.

To examine whether the calculation of AddHRVr episodes actually had the expected advantage over traditional methods, we additionally checked whether the psychological stress variables were associated with mean HRV and mean acceleration level per hour.

The moderating effects of the Levels of Emotional Awareness Scale and resting HRV were examined by adding the main effect of the scores on the Levels of Emotional Awareness Scale or HRV levels—and their interaction with AddHRVr episodes to the models predicting the dependent psychological variables.

We tested our hypothesis using two-tailed significance tests, with *α* = .05. The multilevel models were fit in R, using the packages lme4 [50] and nlme [51]. Figure 2 was created in R using the code provided by Wagenmakers [52].

## Results

### Descriptive Statistics

Of the 51 participants, data from 32 participants could be used for the analyses; 19 participants were excluded, as for 14 insufficient data were available (due to multiple severe artifacts in the data during the calibration), and 5 participants were excluded because the curve estimation regression analysis on the calibration data provided a bad fit to the data (i.e., low level of explained variance (<25 %), no significant relation between movement and RMSSD, or no inverse curve could be fitted). There were no differences between the in- and excluded participants in gender, mean age, and ethnic background. Neither did they differ on the psychological variables, hourly RMSSD, and acceleration levels. Our final sample consisted 32 students, 8 males and 24 females, with a mean age 21.09 (SD = 2.08, range 18–27).

**Fig. 1:**
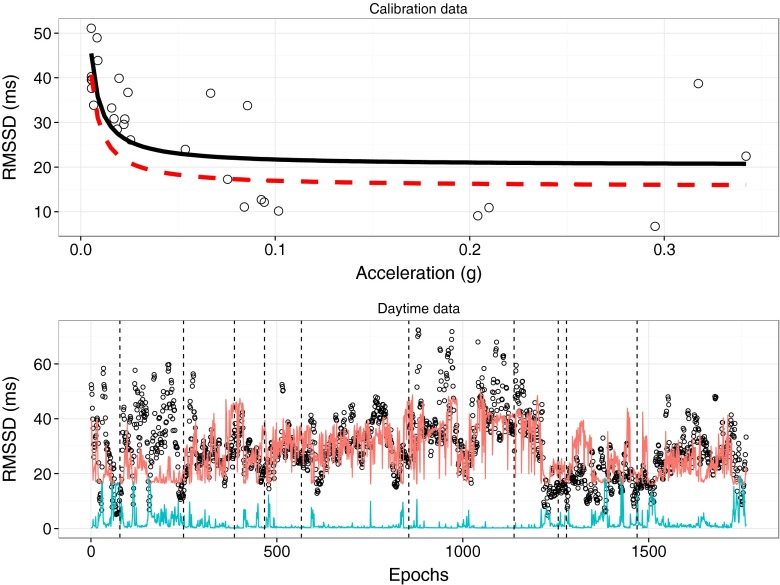
Calibration and daytime data for one participant. In the *upper panel*, the relation between acceleration and RMSSD is depicted. The *solid black line* represents the predicted inverse relation between acceleration and RMSSD, whereas the *dashed red line* depicts the derived threshold for detecting AddHRVr (i.e., predicted RMSSD levels − 2 × SE). In the *lower panel*, the data for the daytime assessments (per 30 s epoch) are shown (scatter; all data points depicted in *open dots*). The *red line* indicates the continuously calculated threshold, and the *open dots below this line* are epochs with AddHRVr decreases. For illustrative purposes, acceleration (multiplied by 50) is also included as the *blue line*. The *dashed black lines* indicate where hourly AddHRVr periods were detected (i.e., a 7.5 min of AddHRVr precedes this line)

Aggregated over all participants, 47 % (SD = 15 %) of the RMSSD data during calibration could be attributed to movement. The average relation between RMSSD and movement during the calibration phase was: Expected RMSSD = 21.56 + .471 / Movement. Average RMSSD (*M* = 44.78, SD = 14.33) and movement (*M* = .06, SD = .03) during the calibration session were similar to levels obtained during the daytime (RMSSD: *M* = 49.63, SD = 22.02; movement: *M* = .06, SD = .02; *t*(31) < 2, *p*s > .05). Figure 1 shows the association between movement and RMSSD for a typical participant.

### Descriptive Data Additional HRV Reduction Episodes and Psychological Variables

Descriptive statistics of all variables are provided in Table 1. A total of 294 hourly episodes were assessed in our study. In 21.8 % (*n* = 64) of the hourly assessments, AddHRVr activity occurred, with a mean of 2 h per participant (SD = 2.17). Nine participants never experienced an AddHRVr episode. Average RMSSD during hours containing an AddHRVr episode (*M* = 36.48, SD = 16.35) was lower than during hours without an AddHRVr episode (*M* = 48.36, SD = 19.49; *B* = 11.65, *t*[278.63] = 5.248, *p* < .0001). There was no difference in average acceleration during the hours with (*M* = .06, SD = .06) or without (*M* = .05, SD = .03) an AddHRVr episode (*B* = −.011, *t*[293.90] = −1.862, *p* = .064).

**Table 1:** Mean, standard deviation (SD), minimum and maximum values of measured variables

	*N*	Mean	SD	Min	Max	Percent
Person level						
Gender	32					75 % female
Age	32	21.09	2.085	18	27	
BMI (kg/m^2^)	31	22.51	2.602	18.19	28.41	
Ethnic background	32					96.9 % Caucasian
LEAS	32	32.38	3.57	27	47	
Measurement level						
AddHRVr	294	2.00	2.17	.00	8.00	21.8 %
RMSSD (ms)	294	45.76	19.45	5.13	121.89	
Acceleration (g)	294	.05	.04	.01	.39	
Implicit NA	294	2.85	1.134	1.00	5.67	
Implicit PA	294	3.34	1.252	1.00	5.67	
Explicit NA	288	1.88	.784	1.00	4.33	
Explicit PA	288	4.00	1.201	1.00	6.00	
Worry episode	294					16.3 %
Worry duration	48	16.52	14.102	.50	45	
Worry intensity	48	3.75	1.296	1	6	
Stress episode	294					6.8 %
Stress duration	20	21.02	15.99	.50	45	
Stress intensity	20	2.30	.657	1	4	

*BMI* body mass index, *LEAS* Levels of Emotional Awareness, *NA* negative affect, *PA* positive affect, *AddHRVr* additional heart rate variability reduction

In addition, on 6.8 % (*n* = 20) of the assessments, one or more stress episodes was reported. On average, participants reported .62 (SD = .87) stress episodes, which in total lasted for about 13.14 min (SD = 22.58). This frequency of reported stressful events is slightly lower compared to previous studies [13, 53]. On 16.3 % (*n* = 48) of the assessments, one or more worry episodes was reported. On average, participants reported 1.50 (SD = 1.81) worry episodes, which lasted in total for about 24.78 min (SD = 33.62), which is comparable to previous studies. Implicit negative affect and implicit positive affect were measured in each episode with a mean of 2.85 (SD = 1.134) for implicit negative affect and a mean of 3.34 (SD = 1.252) for implicit positive affect. The mean of explicit negative affect was 1.88 (SD = .784) and the mean of explicit positive affect was 4.0 (SD = 1.202).

### Relation Between AddHRVr and Stressful Events and Worry Episodes

**Fig. 2:**
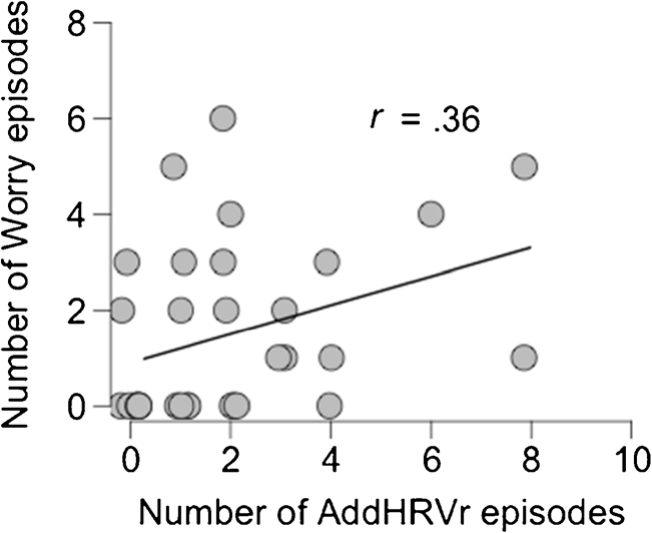
Scatterplot of the association between the total number of additional HRV reduction (AddHRVr) episodes and the total number of worry episodes

Multilevel logistic regression analyses using the two AddHRVr variables (reflecting between- and within-persons variations) as independent variables showed that worry episodes were not associated with AddHRVr-within (*B* = .19, *z* = .42, *p* = .67) or AddHRVr-between (*B* = 1.90, *z* = 1.41, *p* = .16). When purely examining the between-persons association using a Pearson correlation, the total number of AddHRVr episodes was significantly associated with the total number of worry episodes (*r*(30) = .36, *p* = .043—see also Fig. 2).

Stress episodes were not associated with AddHRVr-within (*B* = −.09, *z* = −.14, *p* = .88) or AddHRVr-between (*B* = −.21, *z* = −.16, *p* = .87). Pearson correlation on the aggregated data also showed no between-person association (*r*(30) = .15, *p* = .401). The average hourly levels of RMSSD and movement were not associated with the amount of stress or worry episodes.

Exploratory correlational analyses concerning the different facets of worry (duration and intensity) showed that the association between AddHRVr and worry intensity was marginally significant (*r*(30) = .33, *p* = .06), but the association between AddHRVr and worry duration was not significant (*r*(30) = .27, *p* = .13).

### Relation Between AddHRVr and Explicit and Implicit Affect

**Fig. 3:**
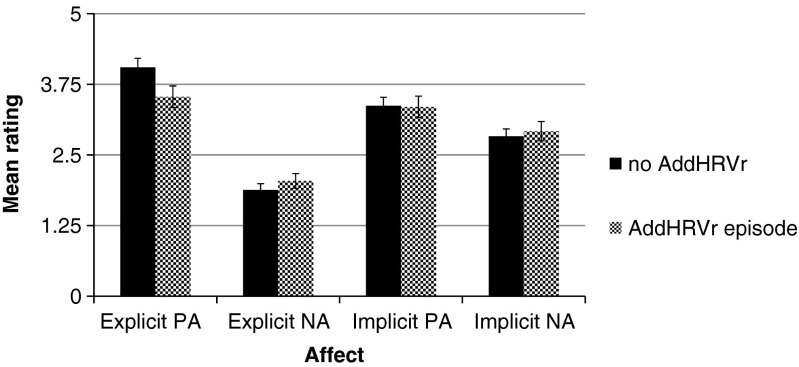
Estimated marginal means (+/− SEM) of the affect ratings for hours without and with an episode of additional heart rate variability reduction (AddHRVr). *PA* = positive affect, *NA* = negative affect

Figure 3 shows the means and standard errors of the affect variables in hours with and without AddHRVr. For explicit positive affect, only within-person changes in the presence of AddHRVr episodes were significantly associated with lower ratings of explicit positive affect (*B* = −.513, *t*[255] = 3.69, *p* < .001). For explicit negative affect, only within-person changes in AddHRVr were marginally associated with higher levels of explicit negative affect (*B* = .15, *t*[255] = 1.72, *p* = .086). Within- and between-person changes in AddHRVr were not associated with implicit positive affect or implicit negative affect (all *p*s > .45).

The average hourly levels of RMSSD and movement were not associated with explicit or implicit affect.

### Moderating Effects of Emotional Awareness and Resting HRV

**Fig. 4:**
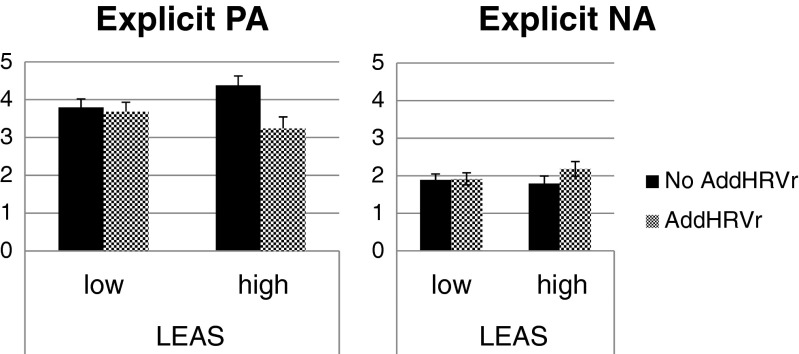
Estimated marginal means (+/− SEM) of the affect ratings for hours without and with an episode of additional heart rate variability (AddHRVr), split by participants low and high in emotional awareness (median split). *PA* = positive affect, *NA* = negative affect, *LEAS* = Levels of Emotional Awareness Scale

As expected, a significant relation between the two proposed moderators, scores on the Levels of Emotional Awareness Scale and resting RMSSD levels, was observed, *ρ* = .423, *p* < .05. Moderation analyses showed that the scores on the Levels of Emotional Awareness Scale significantly moderated the associations between AddHRVr and explicit positive affect (*B* = −.28, *t*[244] = −4.10, *p* = .001) and between AddHRVr and explicit negative affect (*B* = .09, *t*[244] = 2.14, *p* = .03). Figure 4 shows the relation between AddHRVr and explicit positive affect and negative affect, split for individuals low and high in emotional awareness (median split for illustrative purposes). These graphs illustrate that AddHRVr was associated with reduced explicit positive affect and heightened explicit negative affect, but only for participants high in emotional awareness. A similar pattern was observed for resting levels of HRV, where AddHRVr was associated with reduced explicit positive affect and heightened explicit NA, but only for participants with high RMSSD levels during rest.

### Exploratory Analyses

We additionally explored whether AddHRVr episodes were associated with the three specific emotions included in the explicit negative affect scales (explicit positive affect was measured using one item, Happiness). Regarding explicit negative affect, within-person changes in the presence of AddHRVr episodes were significantly associated with explicit Tension (*B* = .41, *t*[255] = 2.62, *p* < .01), but not with explicit Anger (*B* = −.08, *t*[255] = −.74, *p* = .45), or explicit Sadness (*B* = .12, *t*[255] = 1.19, *p* = .23). With respect to sadness, gender differences in the relation between sadness and HRV have previously been found, and we examined whether gender moderated the relation between AddHRVr episodes and sadness, which was the case, *B* = −.92, *t*[254] = −3.18, *p* = .002. In men, AddHRVr episodes were associated with increased sadness (*B* = .91, *p* < .001), which was not apparent for women (*B* = −.01, *p* = .90).

## Discussion

The main purpose of this study was to examine whether it was possible to detect prolonged (i.e., >7.5 min) periods of additional, non-metabolic, HRV reductions in daily life when using a person-specific calibration procedure. After obtaining the relation between movement and HRV during several simple exercises, we were able to detect prolonged periods during which HRV was reduced when there was no movement causing that reduction. Almost half of the hourly assessments that we obtained encompassed at least one such period. The proposed method adds to the existing possibilities to identify shorter moments of additional cardiac activity in daily life [19, 26, 27, 54]. The findings provide a proof of principle, in a healthy convenience sample, that it is possible to distinguish between prolonged metabolic and non-metabolic HRV reductions in daily life, when using a simple calibration session. Researchers interested in making this distinction would only be required to add a short calibration phase to the study protocol, as the calculations can be conducted afterwards if the ECG and movement data are available.

Within-person changes in AddHRVr periods were associated with reduced explicit positive affect and increased explicit negative affect, although the latter was only observed for people that had a greater ability to describe their emotions in a differentiated manner. Furthermore, participants that had more AddHRVr episodes also reported a greater number of worry episodes. These results are in line with studies showing cardiac effects of daily worrying [13, 15] and daily affective states [11]. Yet, we did not observe an association between implicit affect or stressful events and AddHRVr periods. The latter could be due to the rather low number of reported stressful events, which was low when compared to previous studies, and was also lower than the amount of worrying. Perhaps associations between AddHRVr, stressful events, and implicit affect become more apparent in clinically stressed samples.

As AddHRVr episodes were detected in the current data, the proposed algorithm could be used to provide more insight into the hypothesized relations between stress, affect, worries, and physiological stress [29–32]. We are currently pilot testing a newly developed ambulatory system that allows for event-based triggering. We developed a smartphone app that is able to run the algorithm—outlined in this paper—online and this app continuously receives an ECG and acceleration signal from a chest belt, via Bluetooth. If periods of AddHRVr are detected, participants can be prompted to respond to several questions or tasks on the smartphone. Interestingly, this system also allows for stress-reduction interventions to be delivered to participants at exactly those moments that people are experiencing stress (at least physiologically).

Although there was no significant difference in movement between hours containing AddHRVr periods and those without, a trend was apparent. Inspection of Fig. 1 also suggests that AddHRVr periods might be more present when people are moving. It is possible that the AddHRVr detection works best when people are moving. This could be due to the threshold that we used (predicted RMSSD − 2 × SE of RMSSD during calibration), combined with quite some variation in HRV levels when people are moving a lot during the calibration (e.g., Fig. 1, upper panel). If at higher levels of movement the estimation of expected HRV values becomes less precise, and the threshold to detect HRV reductions is quite liberal, chances increase that prolonged AddHRVr reductions are detected. Of course, a longer calibration phase with longer periods of movement could be used to possibly decrease this variance. Or, alternatively, the threshold could be increased. Still, only a trend was observed and we did find that during hours with AddHRVr periods, explicit positive affect was reduced and worry was enhanced, whereas these relations were not observed when examining the raw HRV values, suggesting that using the proposed algorithm has advantages to detect associations between psychological stress and cardiac activity. At this stage, further studies that manipulate the different components of the algorithm (i.e., threshold, duration of the calibration phase, type of HRV parameter used) are warranted.

Although it was not our primary aim, this study is in fact the first to show that resting HRV and levels of emotional awareness are related. This association has previously been predicted based on studies showing that higher levels of emotional awareness [55] and resting HRV [56] are each associated with greater activity in medial prefrontal and rostral anterior cingulate cortex. Here, we show that resting HRV and levels of emotional awareness are indeed positively correlated, providing further evidence for the neurovisceral integration model of emotion (dys)regulation [34, 56]. Both variables moderated the relation between AddHRVr episodes and explicit affect. Only for participants high in resting HRV or emotional awareness did explicit emotions covary with the presence of AddHRVr episodes. An important implication of this moderation is that it highlights the potential benefit of AddHRVr monitoring as a marker of physiological stress in individuals who are lower in emotional awareness. That is, for the latter, individuals self-reported stress or distress may not be a reliable indicator of when they are physiologically stressed in a way that may affect their health, whereas in higher emotional awareness self-reported stress or distress would be a more reliable indicator. Even for the latter individuals, however, AddHRVr monitoring in real time may be useful because it could enable those individuals to be aware of their physiological state of stress if they are busy attending to things other than how they are feeling.

In the exploratory analyses, gender also appeared to moderate the relation between AddHRVr episodes and sadness. In men, AddHRVr episodes were associated with increased sadness but this was not apparent for women. This is in line with previous studies showing gender differences in the relation between sadness and depressive symptoms and resting and circadian HRV levels [57–59].

Several limitations have to be taken into account. We used a small, healthy and young sample of students. It remains to be understood whether AddHRVr periods can be detected in people that have (chronically) lower levels of HRV, such as older samples, or patients suffering from anxiety or mood disorders [60, 61]. We also had to exclude 19 participants, mostly because the quality of the data obtained during the short calibration contained too many artifacts. The excluded participants did not differ from the participants with respect to the psychological and physiological variables. Because of this lack of differences, the artifacts seem to be distributed randomly. Still, it prevents strong conclusions about the generalizability of the findings. Because the calibration phase yields only a few data points, missing values quickly become a problem when creating a personalized algorithm. As there were also participants for whom no algorithm could be created, future studies are warranted to develop and test other algorithms and other calibration procedures. Furthermore, it remains unclear how the algorithm would perform when using other calibration exercises. For example, we used cycling and walking up and down the stairs. Although these exercises can form part of the daily lives of participants, these exercises might not be feasible to conduct at each research facility. Additionally, we used cycling on a stationary bike, which in the flat country of the Netherlands might reflect cycling in daily life to some extent, it remains unclear how the algorithm would work in countries with hills and mountains. Finally, it remains unclear how stable the personalized algorithms are, i.e., what the test-retest reliability is. Thus, although we here provide a method to capture AddHRVr periods in daily life, it remains unclear how this would work in other geographical contexts or when using the same algorithm across several days.

In summary, prolonged non-metabolic reductions in daily HRV levels can be detected using an algorithm that took the person-specific relation between movement and HRV into account. The detected prolonged episodes were associated with increased worries and decreased positive affect. Combined with recent advances in smartphone and sensor technologies, the real-time assessment of episodes of detrimental prolonged cardiac activity is possible and might be a fruitful next step for psychophysiological research.
